# Thoroughbred mare's milk exhibits a unique and diverse free oligosaccharide profile

**DOI:** 10.1002/2211-5463.12460

**Published:** 2018-07-16

**Authors:** Sercan Karav, Jaime Salcedo, Steven A. Frese, Daniela Barile

**Affiliations:** ^1^ Department of Molecular Biology and Genetics Canakkale Onsekiz Mart University Canakkale Turkey; ^2^ Department of Food Science and Technology University of California Davis CA USA; ^3^ Evolve Biosystems, Inc. Davis CA USA; ^4^ Foods for Health Institute University of California Davis CA USA

**Keywords:** free oligosaccharides, Mare's milk, nano LC‐Chip QToF‐MS

## Abstract

The Thoroughbred is among the most valuable horse breeds, and its husbandry is a major industry. Mare's milk plays a major role in the health of neonatal foals. Although the main components of mare's milk are broadly characterized, free oligosaccharides (OS), which possess various bioactivities in many mammalian milks, have not been fully profiled in Thoroughbreds. The aim of this study was to identify and quantify OS in Thoroughbred mare's milk during the first week of lactation, when foals typically consume mare's milk exclusively. A total of 48 OS structures (including isomers and anomers), corresponding to 20 unique compositions, were identified by nano LC‐Chip QToF‐MS and confirmed by tandem mass spectrometry. Neutral OS were the most abundant glycans (58.3%), followed by acidic OS containing Neu5Ac (33.3%), a minor presence of fucosylated OS structures (6.25%) and one structure containing NeuGc (2.1%). Comparison with other well‐characterized mammalian milks revealed that mare's milk shared 8 OS structures with human, bovine, pig and goat milk (i.e., 2 sialyllactose isomers, 3 hexose, LNH, LNT, and OS with the composition 3 Hex‐1 Neu5Ac). Additionally, there were seven unique OS not previously found in other mammal milks. During the first 7 days of lactation, the percentage of neutral and fucosylated OS increased, whereas acidic OS decreased and the total OS concentration ranged from 217.8 mg·L^−1^ to 79.8 mg·L^−1^.

**Abbreviations**
Fuc: fucose
GalNAc: *N*‐acetyl‐galactosamine
Glc: glucose
NeuAc: sialic acid
OS: Oligosaccharides

Human milk represents the richest known source of free oligosaccharides and is comprised of over 200 different structures (up to date, 247 human milk OS have been separated, 162 of which have been characterized) with a 3–20 g·L^−1^ concentration 1, 2. Depending on the species, milk oligosaccharides (OS) are typically composed of 3–10 monosaccharide units, including glucose (Glc), galactose (Gal), *N*‐acetyl‐glucosamine (GlcNAc), *N*‐acetyl‐galactosamine (GalNAc), fucose (Fuc), and sialic acids (NeuAc/NeuGc). Their core units can be either lactose [Gal(β1‐4)Glc] or lactosamine [Gal(β1‐4)GlcNAc] 3. Based on chemical composition, OS are classified as neutral (containing glucose/galactose/GlcNAc/GalNAc/fucose) or acidic (which include the previously mentioned monosaccharides and are further decorated by the sialic acids NeuAc/NeuGc). Although these free glycans are not digestible by neonates, they exhibit a wide variety of biological roles, with potential prebiotic, antimicrobial, anti‐adhesive, and immunomodulatory activity 4. In particular, their ability to promote the growth of beneficial microbes in the gut makes these compounds extremely valuable for human health.

Milk oligosaccharides are also known as glycans and play a significant role in the development of the gut microbiome in the early life of mammals 5. Their structural composition determines the accessibility of carbohydrates for bacteria in the large intestine and selects which taxa dominate the distal gut of neonates 6. Extensive characterization of glycans in the gut has elucidated the role of complex carbohydrates, and the breakdown of this complexity limits the growth of pathogens in the gut 7 and how these structures can alter the growth of the host animal 8. In addition to free glycans, OS can also be found conjugated to lipids and proteins, and similarly act as selective growth‐promoting agents for specific species of *Bifidobacterium* 9. A recent study showed that bovine whey colostrum *N*‐glycans released by the novel enzyme endo‐β‐*N‐*acetylglucosaminidase selectively promoted the growth of *B. longum* subsp*. infantis ATCC 15697* 10.

Understanding the structural complexity of milk glycans is critical to determining their function in the distal gut and may provide insight as to how this abundant component of milk may play a role in the health of the neonate across different mammals. Glycans’ diversity of structures and concentration in milk vary significantly depending on the mammalian species, and even among individuals. For example, bovine, porcine, and goat milk contain 55, 39, and 38 free glycan structures, respectively 11, 12, 13. OS with composition 2Hex‐1NeuAc, 1Hex‐HexNAc‐1NeuAc, 4Hex‐2HexNAc, and 3Hex‐1HexNAc are found in human, bovine, goat, and porcine milk, 4Hex‐1HexNAc‐1NeuAc and many larger fucosylated structures are unique of human milk, whereas 4Hex‐1HexNAc is unique for porcine milk, 2Hex‐2HexNAc is only found in goat milk and 2Hex‐2HexNAc‐1NeuAc is unique for bovine milk. Urashima *et al*. reviewed the OS content of different mammalian milk in detail. Understanding the glycan profile of different mammalian milks is important for discovering and characterizing the biological roles of these structures, in view of targeted product development that is species‐specific.

Equines represent a major working or performance animal throughout the world. The husbandry of equines reaches back to human prehistory, and selective breeding has produced animals with significantly diverse phenotypes. For all equines, consumption of colostrum plays a major role in health outcomes for the neonatal foal. This is, in part, a result of the transfer of immunoglobulins, which are actively taken up by the foal during the first days of life. However, transitional/mature milk certainly also plays a key role in the health of the neonatal foal as in other mammals. Although the composition of free glycans has been described for other breeds 14, it has not been reported for Thoroughbreds. Further, mare's milk is a promising alternative to cow's milk for human infants owing to its low fat content and high abundance of whey proteins, including lactoferrin and immunoglobulins 15. In many countries of Asia and East Europe, mare's milk has been an integral part of the daily diet for centuries, whereas interest in the use of mare's milk for infant feeding is more recent, with efforts led by France and Italy in neonatal intensive care units 16, 17, 18. In this study, we investigated the oligosaccharide content of milk collected from four Thoroughbred mares and described how it changed over the first week of lactation.

## Results and Discussion

### Characterization of Mare's milk oligosaccharides

Although different techniques have been used for identifying OS in milk and other biological samples 19, 20, nano LC‐Chip QToF‐MS is one of the most widely adopted technique because of its inherent accuracy and sensitivity and the ability to resolve multiple isomers for each OS without the need for chemical derivatization 13, 21. Using that instrument, several investigators have characterized OS in human, bovine, and porcine milk 3, 22, 23, whereas studies evaluating mare's milk OS are limited. This study identified and quantified OS in mare's milk during the first week of lactation. A total of 48 structures, including isomers and anomers, of OS corresponding to 20 compositions were detected and confirmed by MS/MS in Thoroughbred mare's milk over the first 7 days of lactation (Table 1).

**Table 1:** Composition and relative abundance of OS in the mare's milk samples analyzed. Relative abundance (%) is expressed as the average of values for milk from four mares, each analyzed in triplicate. Composition is reported as Hex, hexose; HexNAc, *N*‐acetylhexosamine; Fuc, fucose; Neu5Ac, *N*‐acetylneuraminic acid; and Neu5Gc, *N*‐acetylglycolylneuraminic acid. RT is retention time in liquid chromatography. Note: OS *m/z* 531.2159 is double charged

Oligosaccharide composition					Exact mass (calc)	Exact mass (expt)	RT (min)	OS Relative abundance (%)						
Hex	HexNAc	Fuc	Neu5Ac	Neu5Gc				Day 1	Day 2	Day 3	Day 4	Day 5	Day 6	Day 7
3	0	0	0	0	506.1848	506.1854	10.49	1.0215	1.4308	0.6095	0.9547	0.9729	0.8388	0.7089
3	0	0	0	0	506.1848	506.1856	13.94	4.4181	5.4741	3.2733	5.6773	5.1573	5.2809	5.5818
3	0	0	0	0	506.1848	506.1867	25.48	0.1253	0.2190	2.7506	0.1410	0.1410	0.1620	0.1980
3	0	0	0	0	506.1848	506.1873	15.89	0.1483	0.0595	0.1176	0.0394	0.0752	0.0554	0.0471
2	1	0	0	0	547.2113	547.2099	13.69	0.1816	0.1298	0.0903	0.1055	0.1192	0.0908	0.0820
2	1	0	0	0	547.2113	547.2118	14.96	0.3442	0.3946	0.2468	0.3923	0.3572	0.3581	0.3234
2	1	0	0	0	547.2113	547.2122	14.37	0.5536	0.1272	0.2502	0.1159	0.0995	0.0715	0.0659
2	1	0	0	0	547.2113	547.2145	11.14	0.1221	0.0311	0.0702	0.0184	0.0278	0.0173	0.0191
2	0	0	1	0	635.2274	635.2281	18.94	21.5480	17.7825	7.3606	14.6102	15.8106	15.3802	15.3318
2	0	0	1	0	635.2274	635.2328	12.42	0.0474	0.1397	7.4111	0.1725	0.1976	0.3237	0.3078
2	0	0	0	1	651.2218	651.228	18.3	0.0734	0.0483	0.0759	0.0273	0.0265	0.0157	0.0143
4	0	0	0	0	668.2376	668.2384	12.77	0.6473	0.3418	0.1702	0.2528	0.3156	0.1814	0.1424
4	0	0	0	0	668.2376	668.2388	14.42	0.8927	0.8751	0.5181	0.8527	0.7044	0.5164	0.4751
4	0	0	0	0	668.2376	668.241	16.07	0.2425	0.0610	0.4065	0.0563	0.1094	0.0459	0.0387
1	1	0	1	0	676.2534	676.254	19.49	2.3584	0.0951	0.0923	0.0827	0.1292	0.0697	0.0504
3	1	0	0	0	709.2642	709.2643	17.98	0.9538	0.8697	0.5236	0.9781	0.9082	0.8503	0.8014
3	1	0	0	0	709.2642	709.2645	24.87	1.5970	0.8472	0.7997	0.5422	0.5786	0.4353	0.3735
3	1	0	0	0	709.2642	709.2645	14.96	1.0818	1.3143	0.9871	1.3031	1.2064	1.1786	1.0591
3	1	0	0	0	709.2642	709.2652	11.84	12.9499	12.9067	6.0894	9.9517	9.9498	8.3908	6.9171
3	1	0	0	0	709.2642	709.2657	14.2	11.3807	19.6812	18.1737	27.0419	27.3783	31.0953	33.0486
2	2	0	0	0	750.2907	750.2853	16.16	0.1671	0.0736	12.7879	0.0312	0.0733	0.0743	0.0745
2	2	0	0	0	750.2907	750.2885	13.53	0.5731	0.1958	0.1140	0.1392	0.1656	0.1254	0.1452
2	2	0	0	0	750.2907	750.2907	24	0.2206	0.0236	0.0795	0.0063	0.0110	0.0025	0.0000
2	2	0	0	0	750.2907	750.2911	19.55	0.0580	0.0866	0.0414	0.0774	0.0597	0.0729	0.0694
2	2	0	0	0	750.2907	750.2911	12.6	0.6886	0.2472	0.2407	0.4498	0.5382	0.6418	0.6943
3	0	0	1	0	797.2802	797.2804	21.51	1.3695	0.8747	0.5309	0.6153	0.6314	0.4256	0.4442
3	0	0	1	0	797.2802	797.2805	19.44	0.4063	0.1417	0.4121	0.1757	0.1207	0.1403	0.1271
5	0	0	0	0	830.2905	830.2912	13.42	3.1050	0.3951	1.0053	0.1885	1.8018	0.2039	0.1326
2	1	0	1	0	838.3062	838.3057	21.98	1.5229	0.0042	0.9213	0.0016	0.0153	0.0000	0.0000
2	1	0	1	0	838.3062	838.3061	21.5	1.6260	0.0037	0.0000	0.0007	0.0153	0.0000	0.0000
2	1	0	1	0	838.3062	838.3068	19.67	1.6303	0.0145	0.0023	0.0079	0.0207	0.0047	0.0050
4	1	0	0	0	871.3170	871.317	24.87	1.6261	0.8018	0.6293	0.5162	0.5523	0.4032	0.3598
4	1	0	0	0	871.3170	871.3184	14.96	16.5201	22.4439	10.4929	21.1238	19.5844	19.7912	19.6040
3	2	0	0	0	912.3436	912.3428	16.48	0.3713	0.1556	10.3487	0.4798	0.3457	0.3267	0.3186
3	2	0	0	0	912.3436	912.3439	13.67	1.6481	3.1469	1.7005	3.2325	2.5625	2.6408	2.8845
5	0	1	0	0	976.3479	976.3497	11.13	1.4788	1.9633	2.7441	2.1045	2.1414	2.0217	2.0111
3	1	0	1	0	1000.3591	1000.356	24.03	0.2053	0.0300	1.2347	0.0408	0.0504	0.0666	0.0621
3	1	0	1	0	1000.3591	1000.358	21.34	0.1908	0.1631	0.2041	0.5430	0.5062	0.7766	0.8085
4	1	1	0	0	1017.3749	1017.374	12.9	0.1418	0.3402	0.3889	0.7203	0.3650	0.7169	0.5219
2	2	0	1	0	1041.3859	1041.381	23.25	0.0744	0.0057	0.2118	0.0017	0.0052	0.0029	0.0045
2	2	0	1	0	1041.3859	1041.384	24.36	0.0631	0.0010	0.0073	0.0000	0.0034	0.0000	0.0000
2	2	0	1	0	1041.3859	1041.385	19.5	0.6799	0.7978	0.3753	0.8113	0.5925	0.7501	0.7035
4	2	0	0	0	1074.3959	1074.394	19.96	0.0482	0.0300	0.3900	0.0422	0.0460	0.0303	0.0300
4	2	0	0	0	1074.3959	1074.396	17.92	3.5791	4.4022	2.3006	4.5321	4.6143	4.4800	4.6152
4	1	0	1	0	1162.4119	1162.410	24.14	0.2295	0.0931	2.3041	0.0457	0.0896	0.1241	0.0530
4	1	0	1	0	1162.4119	1162.412	24.87	0.8838	0.5358	0.2511	0.4846	0.4865	0.4325	0.3767
4	2	0	1	0	1365.4909	1365.491	24.12	0.0849	0.0517	0.2946	0.1172	0.1605	0.1618	0.1441
1	1	1	0	0	531.2159	531.2146	26.05	0.1097	0.1284	0.1693	0.1777	0.1561	0.1939	0.1872

Among the OS structures identified, some OS are described for the first time in Thoroughbred mare's milk. Urashima *et al*. determined new neutral OS from horse colostrum including Gal(β1‐3)Gal(β1‐4)Glc, Gal(β1‐6)Gal(β1‐4)Glc, and Gal(β1‐3)[Gal(β1‐4)GlcNAc(β1‐6)]Gal(β1‐4)Glc 24, 25 and unusual phosphorylated N‐acetyllactosamine 26. The number of OS identified is higher than reported in the literature so far. Albrecht *et al*. 27 reported in a recent review 37 OS in mare's colostrum, and Difilippo *et al*. 14 reported 16 OS in the mature milk of four breeds, being especially important the difference in the presence/absence of OS type 1. Differences in the findings of these studies can be explained by the diverse methodologies used, mare breeds studied and lower abundance of some OS (especially OS type 1). It is also likely that the untargeted approach used in this study, combined with the high accuracy, sensitivity, and excellent chromatographic resolution obtained by the nano LC‐Chip QToF‐MS technology 24, allowed the detection of all the OS in the samples analyzed.

Neutral OS were the most abundant (58.3%), followed by acidic OS containing Neu5Ac (33.3%), with a minor presence of fucosylated OS structures (6.25%) and only one structure containing NeuGc (2.1%). In comparison, Albrecht *et al*. 27 reported that acidic OS containing NeuAc were the most abundant (54.5%) followed by neutral (43.2%) and fucosylated (2.7%). Difilippo *et al*. 14 reported the same ratio of neutral and acidic structures but did not detect any fucosylated OS in mare's milk.

Thoroughbred mare's milk OS have less structural variety compared with human milk's nearly 200 characterized structures 28; yet, when comparing the overall OS structural typology and diversity, mare milk contains a higher number of OS with structural features that are uniquely found in human milk and are only found at the trace level in bovine milk 11. Additionally, OS in Thoroughbred mare's milk are found in greater array of oligosaccharides (48 structures here identified) compared to what described for porcine milk (39 structures) 12 or goat milk (38 structures) 13. Similarly to human and bovine milk, a few OS structures comprised more than 60% of the total OS, in this case, 3_1_0_0_0, 4_1_0_0_1 LNnP‐I and 3′‐SL. Despite the number of fucosylated structures characterized in mare's milk and in porcine milk being similar, the contribution of these OS to the total was slightly lower (6.25% in mare vs. 9.1% in porcine milk) yet, still higher than that found in bovine milk (1%) 29. Human milk contains both type I (LNT, LNH, LNFP‐II) and type II (LNnT, LNnH, LNFP‐III) OS, whereas bovine milk has been shown to contain predominantly type II core OS with lower amounts of OS type I 30. In contrast, mare's milk contained both type I (LNT, LNH) and type II (LNnT, LNnH) OS in considerable amounts.

### Mare's milk oligosaccharide variation during early lactation

**Figure 1:**
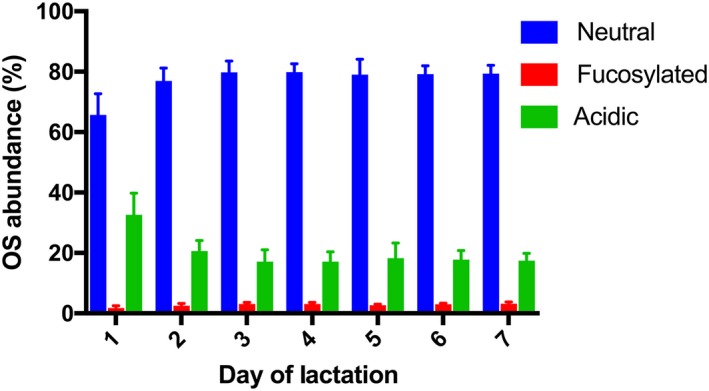
Variation of mare's milk OS type (neutral, fucosylated, acidic) during the first week of lactation by nano LC‐Chip QToF‐MS. Results are expressed as the average ± standard deviation (*n *=* *4) of the relative abundance of each class of OS in milk from four animals.

**Figure 2:**
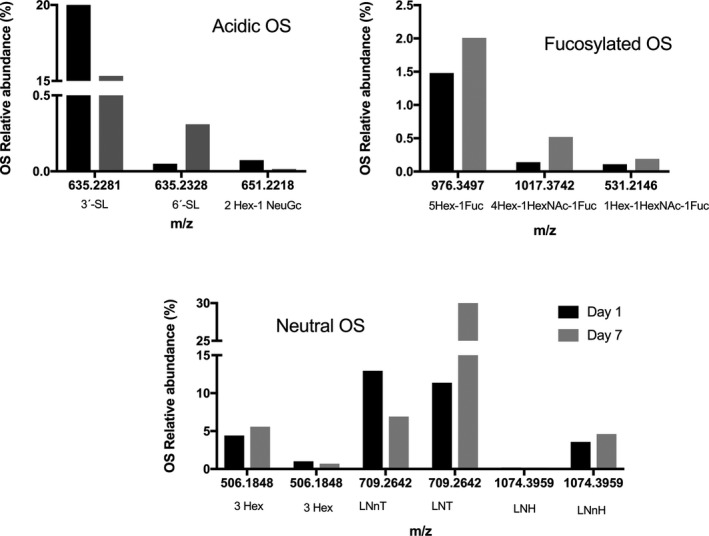
Variation of key OS in mare's milk during the first week of lactation measured by nano LC‐Chip QToF‐MS. Results are expressed as the average ± standard deviation (*n *=* *4) of the relative abundance of each class of OS.

Oligosaccharides class variation was evaluated during the first week of lactation (Fig. 1). Whereas neutral OS increased from Day 1 to Day 2 and remained stable during the rest of period evaluated, acidic OS decreased in the first 2 days and remained stable afterward. Interestingly, there was a modest but noticeable increase in fucosylated OS at Day 3 (from 1.7% to 3.05%), and their abundance remained stable up to Day 7. These OS variations are further explored in Fig. 2, where the most abundant OS of each class are plotted—3′‐SL, the most abundant at Day 1, decreased from 21.6% at Day 1 to 15.3% at Day 7; LNT increased from 11.4% to 33.0%; and the OS with composition 5_0_1_0_0 varied from 1.5% to 2.0%. The single OS structure containing NeuGc decreased with time, with abundance from 0.073% to trace amount (0.014%).

**Figure 3:**
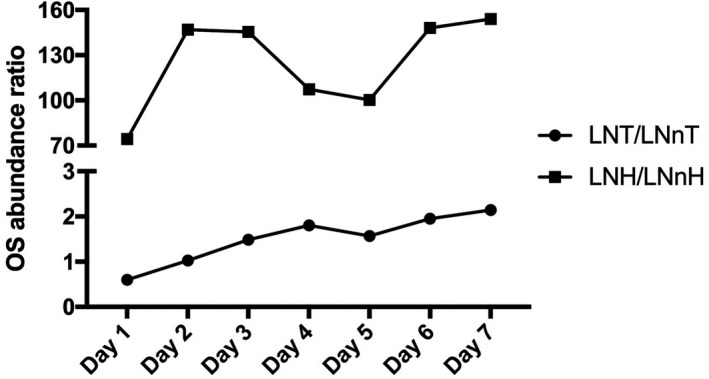
Evolution of LNT/LNnT and LNH/LNnH during lactation analyzed by nano LC‐Chip QToF‐MS.

To evaluate the predominant OS core type, the ratio of LNT/LNnT and LNH/LNnH was calculated along lactation (Fig. 3). At Day 1, LNnT was predominant over LNT (ratio < 1), which was opposite to the ratio of LNH and LNnH (ratio > 1); however, the ratio of type I/type II OS increased with lactation in both comparisons, suggesting mare's milk composition becomes relatively closer to that of human milk as lactation advances as indicated by its higher content of type I OS and the marginal increase in fucosylated OS.

**Figure 4:**
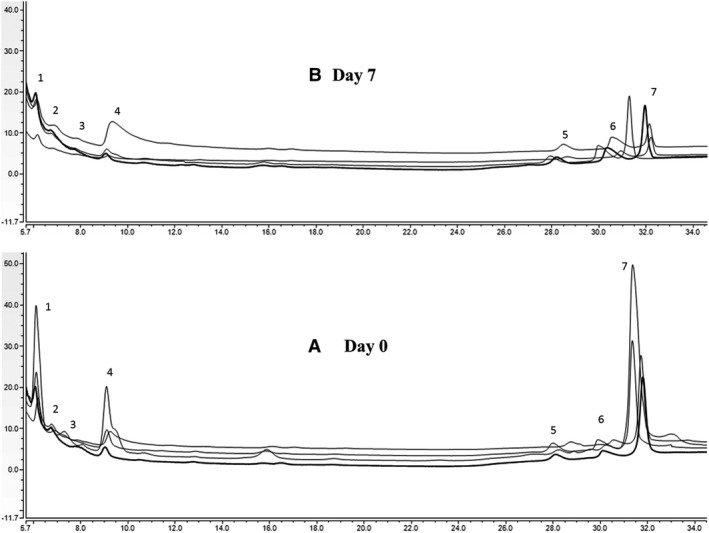
HPAEC‐PAD chromatograms of OS in four mare milks at different lactation stages. (A) Day 0, (B) Day 7. Peak 1, 2Hex‐1HexNAc; Peak 2, LNnT; Peak 3, 3 hexose; Peak 4, LNT; Peak 5, 3′‐SLN; Peak 6, 6′‐SL; and Peak 7, 3′‐SL.

To fully characterize the OS in mare's milk, this dataset was further analyzed by high‐performance anion‐exchange chromatography coupled with pulsed electrochemical detection (HPAEC‐PAD) to measure the concentration of lactose and OS during the first week of lactation. To date, the commercially available OS standards at the necessary purity are scarce, limiting the number of structures quantifiable compared with the OS identified by nano LC‐Chip QToF‐MS. Ten OS, as well as lactose, were monitored for concentration over the first week of lactation (Fig. 4). The most abundant OS were 3′‐SL, 6′‐SLN, and 6′‐SL, whereas for neutral OS, LNT was predominant, followed by LNnT and 3 hexose and 2 Hex‐1HexNAc. The lactose content was in the range 18–24 g·L^−1^, a value lower than that described for human milk (60–65 g·L^−1^) and bovine milk (44–52 g·L^−1^).

The concentration of total OS at lactation Day 1 was 217 mg·L^−1^, whereas it decreased throughout lactation; 117 mg·L^−1^ at Day 4 and 79 mg·L^−1^ at Day 7 (Table 2). Tao *et al*. 11 demonstrated that bovine milk contained significantly higher OS at Day 1 compared with our findings, but the total OS concentrations became similar at Day 7.

**Table 2:** Quantification of OS in mare's milk (mg·L^−1^) during the first 7 days of lactation. Results expressed as average ± standard deviation (*n *=* *3) for each one of the four animals

	[Oligosaccharide] (mg·L^−1^)							
	2 Hex ‐ 1 HexNAc	LNnT	3 Hex	LNT	6′‐SLN	6′‐SL	3′‐SL	Total OS
RT (min)	4.33	5.87	7.79	8.7	26.64	29.13	31.08	
Day 1	0.504 ± 0.401	1.165 ± 1.185	0.971 ± 0.471	8.928 ± 8.728	11.719 ± 4.5	7.126 ± 3.922	187.531 ± 126.126	217.815 ± 131.302
Day 2	0.745 ± 0.48	1.026 ± 0.44	0.871 ± 0.515	6.892 ± 3.982	19.638 ± 7.5	16.848 ± 1.241	105.327 ± 33.333	151.108 ± 35.307
Day 3	0.703 ± 0.406	0.695 ± 0.413	0.751 ± 0.424	8.86 ± 6.09	18.044 ± 5.903	18.737 ± 4.408	79.381 ± 39.553	127.151 ± 43.395
Day 4	0.681 ± 0.255	0.529 ± 0.2	0.975 ± 0.3	6.656 ± 4.217	18.781 ± 3.877	18.189 ± 2.295	71.526 ± 25.97	117.161 ± 30.115
Day 5	0.777 ± 0.594	0.403 ± 0.096	0.827 ± 0.186	8.174 ± 4.6	18.789 ± 6.053	15.75 ± 2.604	50.318 ± 15.632	94.625 ± 23.204
Day 6	0.715 ± 0.29	0.367 ± 0.146	0.648 ± 0.293	7.194 ± 2.638	15.86 ± 5.052	15.635 ± 4.482	48.983 ± 18.914	89.078 ± 26.266
Day 7	0.953 ± 0.87	0.273 ± 0.09	0 ± 0	2.367 ± 2.047	13.984 ± 3.137	13.382 ± 5.789	48.793 ± 37.598	79.753 ± 40.833

When the individual concentrations of specific OS were evaluated, different trends were observed depending on the OS considered. The apparent high standard deviation did not derive from measurement issues, but rather was the result of the natural basal level of OS in the four animals considered. Regardless of the variation in concentration, some trends were observed in all the four animals. For example, while 3′‐SL, LNT, and LNnT continuously decreased with time, 6′‐SL and 6′‐SLN increased, 2 Hex‐1HexNAc remained stable until Day 6, and 3 Hex which was low abundant became undetectable at Day 7 (Table 2). Independently of the individual OS variation, there was a net decrease in the total neutral and acidic OS with time—total acidic OS sharply decreased during the first days while neutral OS remained stable for 6 days. Lactose content increased during the first week of lactation from 18.2 g·L^−1^ at Day 1 to 24.0 g·L^−1^ at Day 7 of lactation. This trend is also observed in human and bovine milk, showing a well‐known increase during the first weeks of lactation 31, 32. However, mare's milk had a lower lactose concentration when compared with other mammal milks 33.

### Comparison of Mare's milk OS with other mammalian milks

Unique OS are synthesized only by certain species (e.g., human milk contains unique fucosylated OS) due to the different genetic, metabolic, and lactation‐specific synthetic pathways 34, 35. This work demonstrated that Thoroughbred mare's milk shares eight OS structures with human, bovine, pig, and goat milk (3′sialyllactose, 6′sialyllactose, 3 hexose, LNnH, LNH, LNT, LNnT, and OS with composition 3 Hex‐1 Neu5Ac), but it also contains seven specific OS not reported in other mammal milks (Table 3). The highest number of shared OS structures is with porcine milk (29), followed by bovine milk (28) and goat milk (26). However, when compared with human milk OS composition, there is a higher number of OS shared between human and Thoroughbred mare milk (19) than between human and porcine (13) 29 or bovine milks (11) 3.

**Table 3:** Oligosaccharides in mare, human, bovine, goat, and porcine milks. OS *m/z* 531.2159 is double charged. New OS structures identified in Mare Milk (in bold)

Mare milk			Presence in other milks			
OS composition	Exact mass (calc)	RT (min)	Human milk	Bovine milk	Goat milk	Porcine milk
3_0_0_0_0	506.1848	10.49	✓	✓	✓	✓
3_0_0_0_0	506.1848	13.94	✓	✓	✓	✓
3_0_0_0_0	506.1848	25.48	✓	✗	✓	✗
3_0_0_0_0	506.1848	15.89	✓	✓	✓	✓
**2_1_0_0_0**	**547.2113**	**13.69**	✗	✓	✓	✓
2_1_0_0_0	547.2113	14.96	✗	✓	✓	✓
2_1_0_0_0	547.2113	14.37	✗	✓	✗	✓
**2_1_0_0_0**	**547.2113**	**11.14**	✗	✓	✗	✓
2_0_0_1_0	635.2274	18.94	✓	✓	✓	✓
2_0_0_1_0	635.2274	12.42	✓	✓	✓	✓
2_0_0_0_1	651.2218	18.3	✗	✓	✗	✓
**4_0_0_0_0**	**668.2376**	**12.77**	✗	✓	✓	✓
4_0_0_0_0	668.2376	14.42	✗	✓	✓	✓
**4_0_0_0_0**	**668.2376**	**16.07**	✗	✓	✓	✗
1_1_0_1_0	676.2534	19.49	✓	✓	✓	✓
**3_1_0_0_0**	**709.2642**	**17.98**	✗	✗	✓	✓
**3_1_0_0_0**	**709.2642**	**24.87**	✗	✗	✓	✓
3_1_0_0_0	709.2642	14.96	✓	✓	✓	✓
3_1_0_0_0	709.2642	11.84	✓	✓	✓	✓
3_1_0_0_0	709.2642	14.2	✗	✓	✓	✓
**2_2_0_0_0**	**750.2907**	**16.16**	✗	✗	✓	✗
**2_2_0_0_0**	**750.2907**	**13.53**	✗	✓	✓	✓
**2_2_0_0_0**	**750.2907**	**24**	✗	✗	✗	✗
**2_2_0_0_0**	**750.2907**	**19.55**	✗	✗	✓	✗
**2_2_0_0_0**	**750.2907**	**12.6**	✗	✓	✗	✗
3_0_0_1_0	797.2802	21.51	✓	✓	✓	✓
**3_0_0_1_0**	**797.2802**	**19.44**	✓	✗	✗	✗
5_0_0_0_0	830.2905	13.42	✓	✓	✗	✗
**2_1_0_1_0**	**838.3062**	**21.98**	✗	✗	✓	✓
**2_1_0_1_0**	**838.3062**	**21.5**	✗	✗	✗	✗
2_1_0_1_0	838.3062	19.67	✗	✗	✗	✗
4_1_0_0_0	871.3170	24.87	✗	✗	✗	✓
4_1_0_0_0	871.3170	14.96	✗	✓	✓	✗
3_2_0_0_0	912.3436	16.48	✗	✓	✗	✓
**3_2_0_0_0**	**912.3436**	**13.67**	✗	✓	✓	✓
5_0_0_1_0	976.3479	11.13	✗	✗	✗	✗
**3_1_0_1_0**	**1000.3591**	**24.03**	✓	✗	✗	✓
3_1_0_1_0	1000.3591	21.34	✓	✓	✗	✓
**4_1_1_0_0**	**1017.3749**	**12.9**	✓	✓	✗	✗
2_2_0_1_0	1041.3859	23.25	✗	✗	✗	✗
**2_2_0_1_0**	**1041.3859**	**24.36**	✗	✗	✗	✗
2_2_0_1_0	1041.3859	19.5	✗	✓	✗	✗
4_2_0_0_0	1074.3959	19.96	✓	✓	✓	✓
4_2_0_0_0	1074.3959	17.92	✓	✗	✗	✓
4_1_0_1_0	1162.4119	24.14	✓	✗	✗	✗
4_1_0_1_0	1162.4119	24.87	✓	✗	✗	✗
**4_2_0_1_0**	**1365.4909**	**24.12**	✗	✗	✓	✓
1_1_1_0_0	531.2159	26.05	✗	✗	✗	✗

## Conclusions

Compositionally, Thoroughbred mare's milk represents a rich source of milk OS for the neonatal foal. A total of 48 OS structures (including isomers and anomers), corresponding to 20 unique compositions, have been identified. Among those, 7 OS were unique for mare milk and were not previously found in other milks. Neutral and, to a certain extent, fucosylated OS increased during the lactation period, whereas acidic OS decreased. The total OS concentration ranged from 217.8 mg·L^−1^ on day 1 to 79.8 mg·L^−1^ on day 7. As with other milk bioactive compounds, these OS are likely to play a significant role on the health of the foal through multifactorial influences, as has been shown for other types of milk. Therefore, it is critical to monitor the composition and abundance of these structures in order to characterize their impact on the developing foal. Overall, OS in Thoroughbred mare's milk are compositionally distinct from other mammalian milk OS, with a higher number of OS shared with human milk than with other domestic animals. These common features may indicate analogous functions across mammals and prompt further studies to assess the importance of milk OS on the development of the gut microbiome and early growth of the foal.

## Materials and methods

### Materials

All solvents used for sample preparation were HPLC‐MS grade (Fisher Scientific, Fair Lawn, NJ). Nonporous graphitized carbon solid‐phase extraction (GCC‐SPE) (2000 μg binding capacity) was purchased from Glygen Corp. (Columbia, MD, USA). Oligosaccharide standards with a minimum purity of 95% (lacto‐*N*‐tetraose, LNT; lacto‐*N*‐neotetraose, LNnT; lacto‐*N*‐hexaose, LNH; lacto‐*N*‐neohexaose, LNnH; acetylgalactosaminyl‐α1,3‐galactose‐β‐1,4‐glucose, 2 Hex – 1 HexNAc; galactose‐α ‐1,3‐galactose‐β‐1,4‐glucose, 3 Hex; 6′‐sialyllactosamine, 6′‐SLN; 3′‐sialyllactosamine, 3′‐SLN; 6′‐sialyllactose, 6′‐SL; 3′‐sialyllactose, 3′‐SL) were purchased from V‐Labs (Covington, LA, USA). Nanopure water (18.2MΩ.cm, 25 °C) was used for the analytical work.

### Sample collection and oligosaccharide isolation and purification

Thoroughbred mare's milk samples were obtained from a commercial Thoroughbred breeding facility (Vacaville, CA, USA). Samples were collected daily from four mares over the first week of lactation and stored at −20 °C until analysis. Milk OS were isolated and purified as previously described, with minor modifications 36. Briefly, frozen milk samples were completely thawed, and a 0.5‐mL aliquot of each sample was mixed with an equal volume of nanopure water and centrifuged at 14 000 × ***g*** in a microfuge for 30 min at 4 °C to remove lipids. The top fat layer was removed, and 4 volumes of chloroform/methanol (2 : 1, vol/vol) were added, vigorously mixed and the resulting emulsion was centrifuged at 4000 × ***g*** for 30 min at 4 °C. The upper methanol layer containing OS was transferred to new tubes, and two volumes of cold ethanol were added. The water/ethanol solution was frozen for 1 h at −30 °C, followed by centrifugation for 30 min at 4000 × ***g*** and 4 °C to precipitate the denatured protein. The supernatant (OS‐rich fraction) was collected and freeze‐dried using a speed vacuum centrifuge.

For nano LC‐Chip QToF‐MS analysis, OS were reduced with NaBH4 1M for 1 h at 60 °C. Once reduced, they were purified from the mixture by solid‐phase extraction using nonporous graphitized carbon cartridges (GCC‐SPE). Prior to use, each GCC‐SPE cartridge was activated with 3 column volumes of 80% acetonitrile (ACN), 0.1% trifluoroacetic acid (TFA, v/v) and equilibrated with 3 column volumes of nanopure water. The carbohydrate‐rich solution was loaded onto the cartridge, and salts and monosaccharides/disaccharides were removed by washing with 10 column volumes (cv) of nanopure water. The OS were eluted with a solution of 40% ACN with 0.1% TFA (v/v) in water and dried in a speed vacuum centrifuge at 35 °C overnight.

### Oligosaccharides characterization by nano LC‐Chip QToF‐MS

Prior to analysis by nano LC‐Chip QToF‐MS, dried OS samples were reconstituted in 100 μL of nanopure water. MS analysis was performed with an Agilent 6520 accurate‐mass quadrupole time‐of‐flight (QToF) liquid chromatography/mass spectroscopy (LC/MS) equipped with a microfluidic nano‐electrospray chip (Agilent Technologies, Santa Clara, CA, USA) as previously described 23. The microfluidic chip contained one enrichment and one analytical column, both packed with graphitized carbon. Chromatographic elution was performed with a binary gradient of 3% ACN/0.1% formic acid in water (solvent A) and 90% ACN/0.1% formic acid in water (solvent B). The column was initially equilibrated with a flow rate of 0.3 μL·min^−1^ for the nanopump and 4 μL·min^−1^ for the capillary pump. The 65‐min gradient was programmed as follows: 0–2.5 min, 0% B; 2.5–20 min, 0–16% B; 20–30 min, 16–44% B; 30–35 min, 44–100% B; 35–45 min, 100% B; and 45–65 min, 0% B. Data were acquired in the positive ionization mode with a 450–2500 mass/charge (*m/z*) range. The electrospray capillary voltage was 1700–1900 V. The acquisition rate was 0.63 spectra/s for both MS and MS/MS modes. Automated precursor selection was employed based on ion abundance, performing up to 6 MS/MS spectra per individual MS when precursor was above ion abundance threshold. The precursor isolation window was selected to be narrow (1.3 *m/z*) to improve accuracy. Fragmentation energy was set at 1.8 V/100 Da with an offset of –2.4 V. Internal calibration was continuously performed by infusing two reference masses: 922.009 and 1221.991* m/z* (ESI‐TOF Tuning Mix G1969–85000, Agilent Technologies). To minimize instrumental variation, diluted samples were spiked with 5 μL of 2‐fucosyllactose 0.02 g·L^−1^, and the results for each OS were normalized against this internal standard.

### Nano LC Chip QTOF data analysis

A list of deconvoluted masses in a range of 450–1500 *m/z* and corresponding to OS was obtained, with all OS compositions confirmed by tandem MS (MS/MS) analysis. The allowed charge states were restricted to single and double species. Following MS/MS identity validation and assessment of reproducible retention times (RT), individual peaks for each OS were automatically integrated using the Targeted Feature Extractor from MassHunter Profinder Version B.06.00 (Agilent Technologies). The RT window allowed for compound matching was restricted to ± 0.5 min and ± 0.25% of the RT at each time point.

### Lactose and oligosaccharides quantification by high‐performance anion‐exchange chromatography coupled with pulsed electrochemical detection (HPAEC‐PAD)

Extracted and purified OS were redissolved in 1 mL of distilled water and diluted 10‐ to 100‐fold in distilled water, filtered through a 0.22‐μm membrane. Aliquots of 25 μL were injected for each analysis. The instrument was equipped with two chromatographic systems allowing simultaneous quantification of lactose and OS quantification; the sample diverted to the correspondent system by a valve installed in the injector system. The chromatographic separation for OS was carried out with a CarboPacPA200 analytical column (3 × 250 mm, Dionex) and a CarboPacPA200 Guard Column (3 × 50 mm, Dionex), eluting at 0.5 mL·min^−1^ with a nonisocratic gradient: 0–10 min 50% B, 10–50 min 45% B–10% **C**. When lactose was quantified, a CarboPacPA10 analytical column (4 × 250 mm, Dionex) and a CarboPacPA10 Guard Column (2 × 50 mm, Dionex) were used, eluting at 1.2 mL·min^−1^ with a nonisocratic gradient: 0–12 min 5% B, 10–25 min 50% B. For both determinations, the columns were equilibrated 5 min with 10% B followed by 10 min with 50% B. Solvent A was deionized water, solvent B was 200 mm NaOH, and solvent C was 100 mm NaAc in 100 mm NaOH.

Simultaneous separation and quantification of 10 different OS were carried out by external calibration ranging from 0.0001 to 0.03 g·L^−1^. From the total of OS quantified, six were neutral (lacto‐*N*‐tetraose (LNT), lacto‐*N*‐neotetraose (LNnT), lacto‐*N*‐hexaose (LNH), lacto‐*N*‐neohexaose (LNnH), acetylgalactosaminyl‐α‐1,3‐galactose‐β‐1,4‐glucose (2 Hex – 1 HexNAc), and galactose‐α‐1,3‐galactose‐β‐1,4‐glucose (3 Hex)) and four were acidic (6′‐sialyllactosamine (6′‐SLN), 3′‐sialyllactosamine (3′‐SLN), 6′‐sialyllactose (6′‐SL), and 3′‐sialyllactose (3′‐SL)). All samples were analyzed in triplicate.

### Statistical analysis

A one‐way ANOVA was performed using SPSS software (SPSS v23.0.0) to evaluate differences in the relative proportion of OS and individual concentrations of OS in mare's milk throughout the first week of lactation. Prior to statistical analysis, normality and homoscedasticity of the data were checked using the Kolmogorov–Smirnov and Levene tests, respectively; all data were normally distributed, and no outliers were identified. Data are presented as least squares means for each lactation time point. Statistical significance was considered when *P* < 0.05.

## Author contributions

SK, JS, and DB conceived and designed the experiments; JS performed the experiments; SAF provided mare milk samples, SK and JS analyzed the data; all authors contributed to writing the manuscript.

## Conflicts of interest

DB is a cofounder of Evolve Biosystems, a company focused on diet‐based manipulation of the gut microbiota. SK is a consultant for Evolve Biosystems. SAF is an employee of Evolve Biosystems.

## Supporting information


**Fig. S1.** OS standard (0.001 g·L^−1^) chromatogram obtained by HPAEC‐PAD. Peak 1, 2Hex‐1HexNAc; Peak 2, LNnT; Peak 3, 3 Hexose; Peak 4, LNT; Peak 5, LNnH; Peak 6, LNH; Peak 7, 6′‐SLN; Peak 8, 3′‐SLN; Peak 9, 6′‐SL and Peak 10, 3′‐SL.
