# Supplementary material for: Thoroughbred mare's milk exhibits a unique and diverse free oligosaccharide profile
Source: FEBS Open Bio. 2018 Jul 16;8(8):1219–29. doi: 10.1002/2211-5463.12460 (PMC6070652; doi:10.1002/2211-5463.12460)

Supplementary Figure 1. OS standard (0.001 g/L) chromatogram obtained by HPAEC-PAD. Peak 1, 2Hex-1HexNAc; Peak 2, LNnT; Peak 3, 3 Hexose; Peak 4, LNT; Peak 5, LNnH; Peak 6, LNH; Peak 7, 6'SLN; Peak 8, 3'SLN; Peak 9, 6'SL and Peak 10, 3'SL

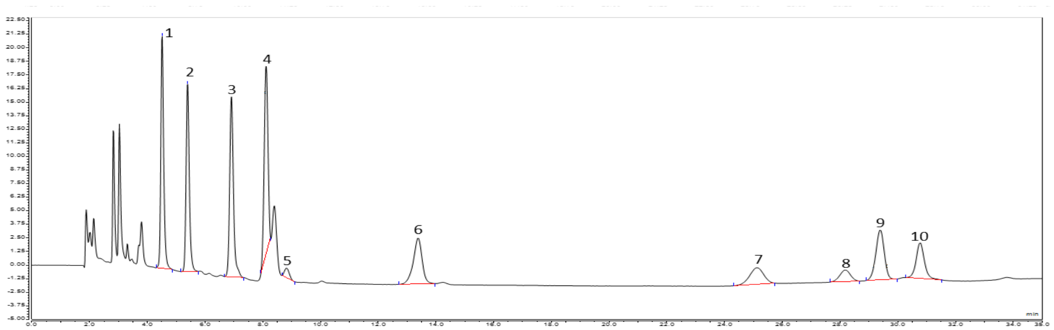

Supplement: Supplementary file 1 — Fig. S1. OS standard (0.001 g·L−1) chromatogram obtained by HPAEC‐PAD. Peak 1, 2Hex‐1HexNAc; Peak 2, LNnT; Peak 3, 3 Hexose; Peak 4, LNT; Peak 5, LNnH; Peak 6, LNH; Peak 7, 6′‐SLN; Peak 8, 3′‐SLN; Peak 9, 6′‐SL and Peak 10, 3′‐SL. [file FEB4-8-1219-s001.pdf]
